# Rural-Urban Differentials in Access to Behaviour Change Communication and Use of Long-Lasting Insecticide-Treated Nets and Artemisinin-Based Combination Therapy in Southeast Nigeria

**DOI:** 10.4314/ejhs.v32i1.7

**Published:** 2022-01

**Authors:** Chidiebere A Nwachukwu, Luke I Anorue, Ijeoma D Ajaero

**Affiliations:** 1 Department of Mass Communication, University of Nigeria, Nsukka Nigeria

**Keywords:** Behaviour Change Communication, Malaria, Rural-urban differentials, Long Lasting Insecticide-Treated Nets, Artemisinin-Based Combination Therapy, Southeast Nigeria

## Abstract

**Background:**

As Malaria continues to take a heavy toll on the life and economy of Nigerians, The National Malaria Elimination Programme uses behaviour change communication (BCC) to promote the use of Long-Lasting Insecticide-treated Nets (LLIN) and Artemisinin-based Combination Therapy (ACT) to combat malaria. This study examined the impact of BCC on the use of LLIN and ACT in Southeast Nigeria.

**Methods:**

A structured questionnaire was used to gather data from 480 respondents in urban and rural communities across five states. Analysis of data was done using percentages, chi-square and logistic regression.

**Results:**

Findings showed weak effect of BCC on LLIN and ACT use despite achieving high (93.75%) exposure. Only 45.1% and 45.7% of the respondents used LLIN and ACT respectively. Urban residents were found to sleep under LLINs and use ACTs more than rural dwellers. Regression results showed that newspapers (OR=1.341) and the Internet (OR=3.216) increased the odds of LLIN use in the rural areas and magazines (OR=1.837) in the urban areas. Television (OR=2.375; P=0.002) and the Internet (OR=6.063; P=0.001) increased the odds of ACT use in the urban areas. Education was found to be a positive predictor of LLIN use in the rural (OR=4.645; P=0.011) and urban areas (OR=6.102) as well as ACT use in the rural (OR=7.268; p=0.002) and urban areas (0R=6.145; P=0.009).

**Conclusion:**

Access to behaviour change communication though very high has not achieved the desired behaviour change. The National Malaria Elimination Programme should produce appropriate messages to address barriers to LLIN and ACT use.

## Introduction

Nigeria is the most malaria endemic country in the world, accounting for 27% and 24% of global malaria morbidity and mortality respectively (1). In addition, malaria costs Nigeria ₦132 Billion in annual economic loss (2). The National Malaria Elimination Programme (NMEP) employs behaviour change communication extensively to persuade Nigerians to sleep under Long Lasting Insecticide-treated Nets (LLINs) and treat malaria with Artemisinin-based Combination Therapy (ACTs) as LLINs and ACTs have been proven efficacious in malaria control.

Studies have shown that exposure to behaviour change communication (BCC) is a positive predictor of LLIN use (3,4,5), and effective in raising public awareness and knowledge of malaria (6,7,8,9,10). This is important because misconceptions about the cause of malaria often result in rejection of promoted malaria control measures (9,11,12) However, there are other variables such as place of residence that affect use of nets. For instance, some studies (13,14,15) found higher ownership of ITNs in rural areas than urban areas. This might be as a result of the fact many nations direct a lot of attention to rural areas in free ITN distribution campaigns. Rural areas suffer from infrastructural deficit, particularly in developing countries (16). However, other studies indicate higher ownership and use of ITNs in urban areas (17, 18).

Moreover, higher education has been found to be a predictor of LLIN use (19,15, 20). One explanation for the readiness of educated people to use LLINs may be that they are less likely to have misconceptions about malaria. Such misconceptions include beliefs that the chemicals in the LLINs would kill unborn children, cause chronic diseases and even sterility (21–23). Complaints of discomfort are in the form of excessive heat and feelings of suffocation associated with net use (24–26). Furthermore, socioeconomic status can also affect adoption of LLIN. Those in the upper socioeconomic quintiles have been reported to be more likely to sleep under nets (27–30)

Whenever malaria prevention fails, attention turns to treatment. Both the World Health Organisation (WHO) and NMEP favour Artemisinin-based combination therapy for malaria treatment (31,32). However, studies have indicated low utilization of ACTs in many places (33–36). The poor utilization of ACT may be traced to competing treatment regimens employed by people. For instance, some studies (37,38, 33) have found high reliance on herbs for malaria treatment among their study populations. Even where people use drugs, the tendency in many places has been to “mix drugs” (39,38) which is the act of combining doses of different drugs for malaria treatment, a practice that is discouraged in the malaria control media campaigns.

Many studies have focused on the impact of BCC on the use of insecticide-treated nets, but there is a dearth in studies that examine both the use of nets and ACTs (32). This study seeks to fill the gap by examining the extent to which Southeast Nigeria residents were exposed to behaviour change communication on malaria and how it has influenced use of LLINs and ACTs for malaria control.

## Methods

The study was a survey of 15 rural and urban communities across the five states that make up the Southeast geo-political region of Nigeria which include Abia, Anambra, Ebonyi, Enugu and Imo states. The study made use of 480 respondents. The sample size was derived from the population of the five states using the Wimmer and Dominick Online sample size calculator.

Data were collected by means of a structured questionnaire containing both open-ended and closed-ended questions. Two dependent/outcome variables were used in the study. These were the use of long-lasting insecticide-treated nets (LLINs) and Artemisinin Combination Therapy (ACT). The respondents were asked if they slept under LLINs the night preceding the survey, and if they used ACT for malaria treatment. For each of the outcome variables, their responses were categorized into 1=yes if they use any of LLIN or ACT and 0= no if they did not use any of LLIN or ACT.

The major independent variable used in the study was exposure to mass media messages on malaria through media channels such as television, radio, magazines, newspapers, the internet and billboards. Respondents who were exposed to each channel were coded 1 (yes) while respondents not exposed to any particular media channel were coded 0 (no). Furthermore, the study made use of individual- level explanatory variables such as gender (male/female), age (less than 30, 30–49, 50+years), education (none/primary, secondary, tertiary), marital status (never married, married, widowed/divorced), employment status (unemployed/student, farmer/businessman, civil servant), income level (up to ₦20,000, ₦21,000-₦50,000, more than ₦50,000).

The data were analyzsed by means of the Statistical Package for the Social Sciences (SPSS). Percentages were used to describe the study population and exposure to malaria messages by respondents. Percentages were also used to describe the use of insecticide-treated nets and ACT. The results of these descriptive analyses were presented in tables. Finally, logistic regression was used to estimate the factors that predict use of insecticide-treated nets and Artemisinin combination therapy among the study population. The regression coefficients of the independent variables are expressed as Odds Ratio (OR). With respect to the coefficients, a variable with Odds Ratio greater than 1.00 indicated that the variable increases the likelihood of ITN/ACT use while a decreased likelihood of ITN/ACT use is implied where the Odds Ratio is less than 1.00. Four regression models were generated for the study. Model 1 shows the use of ITNs in rural areas while Model 2 shows the use of ITNs in urban areas. Model 3 and Model 4 show the use of ACT in rural and urban areas respectively.

## Results

A total of 450 (93.75%) of the respondents reported having been exposed to mass media messages on malaria. Specifically, 93.18% and 94.88% of urban and rural respondents respectively reported being exposed to the media messages. Radio and television were the dominant sources of information on malaria with 79.2% of the respondents reporting having been exposed to the messages via radio and 51.9% through television. Out of the 450 (93.75%) respondents exposed to media messages, 402(89.3%) reported owning at least one LLIN. However, 203(45.1%) reported sleeping under LLINs the night preceding the survey. This comprised 46.74% of urban and 38.03% rural respondents. Furthermore, 206 (45.7%) respondents reported using ACTs for malaria treatment. This comprised 51.91% and 32.56% of urban and rural respondents respectively. Many respondents reported treating malaria with herbal medicine, non-ACT drugs and mixed drugs.

Bivariate/chi-square analyses in Table 1 reveal variations in the impact of demographic variables on access to mass media messages and use of LLINs and ACT for malaria control. Apart from gender (p=0.035) and marital status (p=0.033), the other variables did not differ significantly in their impact on access to media. Similarly, there was no significant difference in the impact of demographic variables on LLIN use except education (p=0.000), employment (P=0.000) and states of residence (P=0.000). It was observed for instance that the use of LLIN increased with rise in educational status. With respect to the use of ACT, there was statistically significant difference in the impact of the variables on ACT use with the exception of gender.

**Table 1:** Bivariate/Chi-square analyses of access to malaria information and use of insecticide- treated nets and Artemisinin combination therapy

Spatio-demographic characteristics	Mass Media			ITN			ACT		

	yes	%	sig.	yes	%	sig.	yes	%	sig.
Gender									
Male	246	96.09	0.035	105	42.00	0.701	111	43.70	0.809
Female	204	91.48		98	43.75		95	42.60	
Age Group									
Less than 29years	113	91.87	0.507	52	43.70	0.317	68	55.74	0.000
0–49years	191	95.02		92	46.00		94	47.24	
50years +	146	94.19		59	38.06		44	28.21	
Marital Status									
Never Married	100	90.91	0.033	46	43.40	0.987	59	53.64	0.000
Married	304	95.90		134	42.54		137	43.49	
Divorced	46	88.24		22	43.14		10	20.00	
Education									
Primary	83	90.22	0.224	23	25.00	0.000	9	9.89	0.000
Secondary	127	94.07		50	37.59		52	38.24	
Tertiary	240	95.24		130	52.21		145	58.00	
Employment									
Unemployed/student	130	94.20	0.352	57	41.61	0.000	62	44.60	0.000
Farmer/businessman	162	92.05		60	34.88		57	32.39	
Civil Servant	158	9.95		86	52.12		87	53.70	
Income									
Up to 20,000	140	90.91	0.131	64	41.83	0.817	67	43.79	0.000
21,000–50,000	177	94.15		79	42.47		63	33.69	
51,000 +	119	96.75		55	45.45		73	59.35	
States									
Enugu	105	92.11	0.430	28	24.56	0.000	31	26.96	0.000
Anambra	101	91.82		61	56.48		64	58.72	
Imo	76	93.83		26	32.50		31	39.24	
Ebonyi	76	97.44		64	82.05		39	50.00	
Imo	92	95.83		24	25.53		41	42.71	
Place of Residence									
Urban	246	93.18	0.437	122	46.74	0.056	136	51.91	0.000
Rural	204	94.88		81	38.03		70	32.56	

Regression analyses were further used to underscore the magnitude of the impact of mass media messages and the demographic variables on the use of LLINs and ACTs.

Table 3 shows regression results of socioeconomic predictors of net and ACT use in the rural and urban areas. Data in model 1 indicate that marital status, education, higher socioeconomic status, being civil servants and farmers increased the odds of net use in the rural areas. For instance, Females were found to be 1.5 times (OR=1.545) likely to sleep under the net relative to males. Those with tertiary education (OR=4.645; P=0.011); and residents of Anambra (OR-8.425; P=0.001); Imo (OR=10.684; P=0.001) and Ebonyi (OR=12.678; P=0.000) were significantly likely to sleep under nets. In the urban areas (model 2), the divorced/widowed, the educated, civil servants and residents of Anambra and Ebonyi states had increased odds of sleeping under nets. Model 3 shows that secondary education (OR=2.951) and tertiary education (OR=7.268; P=0.002) increased the likelihood of ACT use in the rural areas. In addition, data show that rural residents of Anambra, Imo, Ebonyi and Abia states were significantly likely to use ACT relative to residents of Enugu. Among urban dwellers, model 4 indicates that those with tertiary education (OR=6.145; P=0.009) had significantly increased odds of using ACT for malaria treatment. In addition, divorced/widowed people, farmers/businessmen, civil servants and those with higher income were associated with increased odds of ACT use in the urban areas.

**Table 3:** Regression results of socio-economic predictors of LLIN and ACT use

Variables	Model 1(LLIN) Rural	Model 2(LLIN) Urban	Model 3(ACT) Rural	Model 4(ACT) Urban
Gender				
Male (RC)	1.000	1.000	1.000	1.000
Female	1.545	0.764	0.794	0.891
Age				
Less than 29 yrs(RC)	1.000	1.000	1.000	1.000
30–49 years	0.435	0.996	0.633	0.425
50+ years	0.894	1.128	0.498	0.395
Marital status				
Never married(RC)	1.000	1.000	1.000	1.000
Married	0.677	1.71	0.705	1.729
Divorced/widowed	0.497	2.446	0.129	2.782
Education				
Illiterate/primary(RC)	1.000	1.000	1.000	1.000
Secondary	1.646	2.631	2.951	3.129
Tertiary	4.645*	6.102*	7.268**	6.145**
Employment status				
Unemployed/student(RC)	1.000	1.000	1.000	1.000
Farmer/businessman	3.023	1.112	0.919	1.422
Civil servant	2.430	1.474	0.905	1.314
Income				
Up to ₦20,0009(RC)	1.000	1.000	1.000	1.000
21–50,000	0.895	0.637	0.622	0.571
51,000+	1.691	0.548	3.675*	1.074
States				
Enugu(RC)	1.000	1.000	1.000	1.000
Anambra	8.425**	1.951	4.761*	1.663
Imo	10.684**	0.492	4.250*	0.412
Ebonyi	12.678***	5.689**	5.511*	1.327
Abia	1.655	0.690	4.075*	0.596

* p<0.05

** p<0.001

*** p<0.000

Table 4 shows the regression results of the combined effects of mass media and socioeconomic variables on net and ACT use. Data in model 1 show that while television significantly decreased the odds of net use among rural dwellers, newspapers, magazines and the internet increased the odds. In addition, tertiary education (OR=5.005; P=0.018); higher income, being farmers/businessmen positively predicted use of nets in the rural areas. In the urban areas, model 2 shows that all mass media negatively predicted net use except the magazine and internet. The married, divorced/widowed and tertiary education (OR=7.574; P=0.009) were correlated with increased odds of net use among urban dwellers. Model 3 in Table 4 further shows the combined effects of mass media and socioeconomic characteristics on ACT use in the rural areas. As can be observed, access to radio (OR=2.078) increased by 2.07 times the odds of using ACT in the rural areas compared to television (OR=2.375); and the Internet (OR=3.505). Tertiary education (OR=6.921; *p<0.006)*, being residents of Anambra state (OR=5.979; *p<0.012)*; Imo state (OR=8.577; *p<007)*, Ebonyi state (5.334; *p<0.019)* and Abia state (OR=3.446;*p<0.019*) all significantly predicted increased likelihood of ACT use for malaria treatment in the rural areas. In the urban areas, as shown in model 2 only access to billboard messages (2.888; *p<0.011)* and tertiary education increased the odds of ACT use.

**Table 4:** Regression results of combined mass media and socioeconomic predictors of LLIN and ACT use

Variables	Model 1(LLIN) Rural	Model 2 (LLIN) Urban	Model 3 (ACT) Rural	Model 4 (ACT) Urban
Mass media				
Radio: No (RC)	1.000	1.000	1.000	1.000
Yes	0.615	0.506	2.078	1.011
Television: No (RC)	1.000	1.000	1.000	1.000
Yes	0.153**	0.615	2.375	0.857
Newspapers: No (RC)	1.000	1.000	1.000	1.000
Yes	1.809	0.754	0.287	0.448
Magazines: No (RC)	1.000	1.000	1.000	1.000
Yes	1.385	1.685	0.087	1.011
Billboards: No (RC)	1.000	1.000	1.000	1.000
Yes	0.719	0.844	0.685	2.888*
Internet: No(RC)	1.000	1.000	1.000	1.000
Yes	3.355	1.079	3.505	1.095
Gender:				
Male (RC)	1.000	1.000	1.000	1.000
Female	1.713	0.708	0.802	0.920
Age				
Less than 29 years(RC)	1.000	1.000	1.000	1.000
30–49 years	0.291	0.946	0.865	0.380*
50 years +	0.473	1.276	0.726	0.322*
Marital status				
Never Married (RC)	1.000	1.000	1.000	1.000
Married	0.938	2.183	0.554	1.869
Divorced/Widowed	0.656	2.934	0.129	3.160
Education				
Primary Education(RC)	1.000	1.000	1.000	1.000
Secondary Education	1.359	2.973	3.226	3.523
Tertiary Education	5.005*	7.574**	6.921**	7.099**
Employment status				
Unemployed/student(RC)	1.000	1.000	1.000	1.000
Farmer/businessman	3.684*	1.088	0.785	1.574
Civil Servant	2.415	1.385	1.015	1.412
Income level				
Up to 20,000(RC)	1.000	1.000	1.000	1.000
21,000–50,000	1.352	0.652	0.555	0.528
51,000 +	4.550	0.619	2.795	0.946
States				
Enugu (RC)	1.000	1.000	1.000	1.000
Anambra	13.717**	1.643	5.979*	1.800
Imo	13.730**	0.438	8.577**	0.412
Ebonyi	18.659***	6.559**	5.344*	1.871
Abia	2.480	0.601	3.446*	0.728

* p<0.05

** p<0.001

*** p<0.000

## Discussion

There was high exposure to behaviour change communication on malaria prevention in Southeast Nigeria with 93.75% of the respondents having been exposed to the messages. This agrees with other studies that reported high exposure to malaria messages (,^3^,^5^). The most important media were radio and television. The importance of radio and television in malaria campaigns has been well reported (18,5). More urban dwellers (n=246) were exposed to the messages than rural dwellers (n=204).

Despite high exposure to BCC the use of nets and ACT was low in both urban and rural areas. Only 46.7% of urban and 38.03% of rural dwellers slept under LLINs the night before the survey. This low utilization of LLINs has been reported in other studies (13, 14). Similarly, 32.5% of rural dwellers reported treating malaria with ACTs relative to 51.9% in the urban areas. Other studies have found higher use of ACTs in the urban areas. Despite this, the figure is low in view of the 100% compliance with ACT use being promoted by the NMEP. The regression analysis of socioeconomic predictors of LLINs and ACTs showed that post-primary education was correlated with increased odds of LLIN and ACT use with tertiary education significantly increasing the odds in both urban and rural areas. This is consistent with other studies (19, 33). The low utilization of LLIN and ACT indicates weak effect of mass media messages. This differs from a study in Myanmar (40).

The low utilization of LLINs and ACTs despite high exposure to BCC indicates that health communication alone is not enough to get people to use ACTs and nets. Health communication will not compensate for lack of funds to purchase ACTs and LLINs. The Government can help by subsidizing the cost of ACTs and LLINs to make them affordable to the masses. In addition, health communicators should produce health messages in vernacular and complement mass media messages with interpersonal communication by Community Health Workers.

## Figures and Tables

**Table 2:** Regression results of mass media predictors of LLIN and ACT use

Variable	Model 1(LLIN) Rural	Model 2(LLIN) Urban	Model 3(ACT) Rural	Model 4(ACT) Urban
Radio				
No (RC)	1.000	1.000	1.000	1.000
Yes	0.643	0.866	1.579	0.930
Television				
No (RC)	1.000	1.000	1.000	1.000
Yes	0.511*	0.746	2.735**	1.133
Newspapers				
No(RC)	1.000	1.000	1.000	1.000
Yes	1.341	0.877	0.455	0.696
Magazines				
No(RC)	1.000	1.000	1.000	1.000
Yes	1.186	1.837	0.100	0.989
Billboards				
No(RC)	1.000	1.000	1.000	1.000
Yes	0.791	0.712	1.105	2.113*
Internet				
No(RC)	1.000	1.000	1.000	1.000
Yes	3.216*	0.973	6.063**	1.122

* p<0.05

** p<0.001

*** p<0.000
